# Methacrylated Silk Fibroin Additive Manufacturing of Shape Memory Constructs with Possible Application in Bone Regeneration

**DOI:** 10.3390/gels8120833

**Published:** 2022-12-16

**Authors:** Alessio Bucciarelli, Mauro Petretta, Brunella Grigolo, Laura Gambari, Alessandra Maria Bossi, Francesco Grassi, Devid Maniglio

**Affiliations:** 1 Laboratorio RAMSES, IRCCS Istituto Ortopedico Rizzoli, Via di Barbiano 1/10, 40136 Bologna, Italy; 2 RegenHU SA, Z.I. du Vivier 22, 1690 Villaz-St-Pierre, Switzerland; 3 Department of Biotechnology, University of Verona, Strada Le Grazie 15, 37134 Verona, Italy; 4 Department of Industrial Engineering, BIOtech Research Center, University of Trento, Via delle Regole 101, 38123 Trento, Italy

**Keywords:** silk fibroin, Sil-MA, 3D printing, additive manufacturing, tissue engineering, bioprinting, bone tissue engineering

## Abstract

Methacrylated silk (Sil-MA) is a chemically modified silk fibroin specifically designed to be crosslinkable under UV light, which makes this material applicable in additive manufacturing techniques and allows the prototyping and development of patient-specific 2D or 3D constructs. In this study, we produced a thin grid structure based on crosslinked Sil-MA that can be withdrawn and ejected and that can recover its shape after rehydration. A complete chemical and physical characterization of Sil-MA was first conducted. Additionally, we tested Sil-MA biocompatibility according to the International Standard Organization protocols (ISO 10993) ensuring the possibility of using it in future trials. Sil-MA was also tested to verify its ability to support osteogenesis. Overall, Sil-MA was shown to be biocompatible and osteoconductive. Finally, two different additive manufacturing technologies, a Digital Light Processing (DLP) UV projector and a pneumatic extrusion technique, were used to develop a Sil-MA grid construct. A proof-of-concept of its shape-memory property was provided. Together, our data support the hypothesis that Sil-MA grid constructs can be injectable and applicable in bone regeneration applications.

## 1. Introduction

Hydrogels are three-dimensional (3D) polymeric continuous networks able to entrap a large amount of water (up to 99% of the total weight). They do not dissolve in water, and they maintain their shape due to chemical or physical crosslinking or the entanglement of their polymer chains [1,2,3,4].

In the particular case of bone tissue engineering (BTE), hydrogels have several advantages: their mechanical properties and degradation can be tuned according to the crosslinking degree; they can provide nutrients because of their ability to swell, incorporating liquids from outside; they can provide an environment suitable for endogenous cell growth. Moreover, hydrogels are absorbable, and they have excellent integration with surrounding biological tissues, limiting the possibility of inflammatory or immune responses [5].

Hydrogels can be formed by both synthetic and natural derived polymers. Natural polymers have the advantage of being similar to native tissue composition, showing overall better properties compared to their synthetic counterparts, such as low or no cytotoxicity and increased biocompatibility and biodegradability. Among the various natural biopolymers available (i.e., chitosan [6], keratin [7], alginate [8], agarose [9], cellulose [10]), silk fibroin (SF) is interesting because of its high mechanical strength, which makes it a good candidate in the case of structural requirements [11].

SF is the internal protein of silk fiber and is extracted from it as an aqueous solution (regenerated silk fibroin, rSF) by chemical processing [12,13] which contributes also to the removal of the immunogenic sericin. SF is biocompatible [14,15], bioresorbable [16,17], and, when used in the form of gel and film, it is transparent [18,19,20]. Starting with rSF, different procedures have been used to develop hydrogels both by physical and chemical crosslinking.

Physical crosslinking is obtained by inducing the transition of the secondary structure from random coil to β-sheet, which implies the formation of intramolecular hydrogen bonding. Instead, in the chemical crosslinking, the stability is ensured by the formation of covalent bonding and, consequently, a three-dimensional (3D) continuous network [21]. Chemical crosslinking can be obtained using two approaches. The first consists of promoting the formation of dityrosine and trityrosine crosslinking by the use of different agents with or without an external stimulus (ruthenium and ammonium persulfate and UV irradiation [22], riboflavin and UVA irradiation [23], hydrogen peroxide and horseradish peroxidase [24], genipin in presence of humidity [25,26]). The alternative approach is based on the chemical modification of the main SF chain to add reactive moieties that can be activated by external stimuli, like UV light, able to trigger a condensation reaction.

Sil-MA (methacrylated silk) is a chemically modified SF initially designed as ink for 3D fabrication, to be used in digital light processing (DLP) and stereolithography (SLA) 3D printing [27,28]. Sil-MA photocrosslinking is achieved by modifying the lysine side groups of the protein, adding a methacrylic functionality, which can generate stable chemical crosslinking bonds upon the addition of a photoinitiator and UV-exposure. Compared to other previously developed silk modification strategies for photocrosslinking [29,30,31], the main advantages is that the entire process is conducted in water, making it attractive for TE applications.

The resulting material is a hydrogel that can be used for 3D fabrication, realizing custom-shaped structures designed by CAD and printed using light-based 3D technologies. This makes the overall technique able to develop patient-specific devices, based on patient X-rays and magnetic resonance imaging, as widely discussed in the literature [32,33].

Since its development in 2018, Sil-MA has been widely used as a base material for different architectures, including, but not limited to, bone and cartilage scaffolds [32,33,34,35,36], nanoparticles for molecular recognition [37], and sealant for orthodontic applications [38]. The use of Sil-MA as an injectable hydrogel has been only recently introduced in the case of spinal cord injury [39]. The proposed strategy is crosslinking after injection to form the gel in-situ, allowing its stabilization in the injection site [38,39]. This has the disadvantage that the possibility of developing complex structures obtainable by 3D-printing is lost, since the whole injected solution is turned into hydrogel, a fact that can also alter the mechanics at the implant site.

In this work, we adapted a previously developed Sil-MA protocol [27] to a DLP projection and pneumatic extrusion technique that allowed us to print a thin Sil-MA 3D construct capable, once folded, of fully recovering its shape when rehydrated. Sil-MA was produced starting from the raw cocoons and characterized by Fourier Transform Infrared Spectroscopy (FTIR), UV–vis spectroscopy and nuclear magnetic resonance (NMR) to confirm and quantify the methacrylic groups. The rheology of Sil-MA gels and their swelling in water were quantified with different crosslinking times. An initial in vitro biological evaluation using a human lung fibroblast (MRC5) cell line was conducted on the Sil-MA verifying its biocompatibility under the international standard organization standards (ISO 10993). An additional experiment was conducted using human-adipose-derived stem cells (ADSCs) to prove if Sil-MA can support osteogenesis. Finally, we developed a prototypical grid structure based on Sil-MA to perform a withdraw and release assay, demonstrating the shape recovery ability after the ejection from an insertion needle and following re-hydration.

## 2. Results and Discussion

### 2.1. Structural Characterization

**Figure 1:**
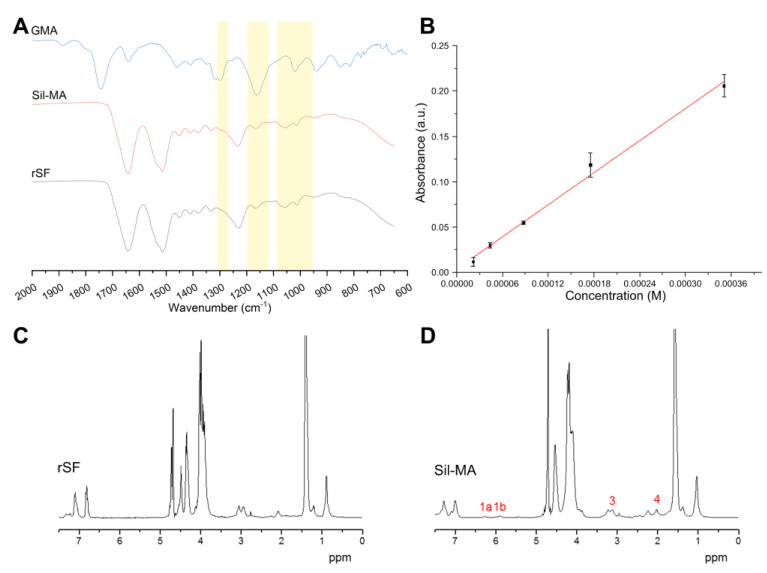
(**A**) FTIR-ATR spectra of regenerated silk fibroin (rSF) and methacrylated silk fibroin (Sil-MA); as reference, the spectrum of glycidyl methacrylate (GMA) was reported. Several modifications (indicated by yellow areas) of the Sil-MA spectra compared to the rSF spectra could be attributed to the presence of functional groups. (**B**) Calibration curve done using β alanine and the TNBS assay and used to determine the percentage of functional groups in Sil-MA. (**C**,**D**) NMR spectra of (**C**) rSF and (**D**) Sil-MA; the peaks present in Sil-MA but not in rSF are numbered and highlighted in red; they confirm the functionalization, except for peak 3, which is the lysine contribution.

The presence of functional groups and the protein secondary structure was qualitatively evaluated by infrared spectroscopy (FTIR−ATR). In Figure 1A, a comparison between the spectra of Sil-MA and regenerated fibroin SF (rSF) is shown. In addition, the spectrum of glycidyl methacrylate (GMA) solution is reported as reference, to verify if peaks of GMA could be recognized in Sil-MA. Small variations in the spectra can be attributed to the presence of the functional groups, in accordance with previously published works [27,34]. The presence of the functional group was shown by the modification of the Sil-MA spectrum compared to the rSF spectrum. Modifications were present at 1265 cm^−1^ (CHOH stretching), 1165 cm^−1^ (CH_2_ wagging stretching), and between 1100 cm^−1^ and 950 cm^−1^ (CH bending of out-of-plane vinyl groups). Using the calibration curve of Figure 1B and the linear regression (Equation (1)), the calculated degree of substitution (DS) for the produced Sil-MA was DS (%) = 33.1%. A similar result was obtained by the NMR analysis comparing the Sil-MA and the rSF spectra, which shows many overlapping signals that can be attributed to the protons of the amino acids of which the protein is composed (alanine, glycine, serine, and other minor contributions). Comparing rSF (Figure 1C) and Sil-MA (Figure 1D) spectra, all CH_3_ and CH_2_ signals were shifted, while the signals from protons produced by aromatic amino acids remained unchanged. The reason for this is difficult to understand, but we can speculate it is due to a different LiBr/D_2_O ratio or a different chain folding induced by methacrylation. In the Sil-MA spectrum, the weak signals of methacrylate groups, the methacrylate vinyl group (1a and 1b in Figure 1D), and the methyl group (4, Figure 1D) could be discerned. The peak labeled with number 3 in Figure 1D is the lysine signal. This signal was integrated after normalization with respect to the signal at 1 ppm to calculate the degree of GMA substitution, which resulted in DS (%) = 33% in accordance with the TNBS assay and previously published results [27,34].

(1) A=589.225C+0.004

### 2.2. Physicochemical Characterization

**Figure 2:**
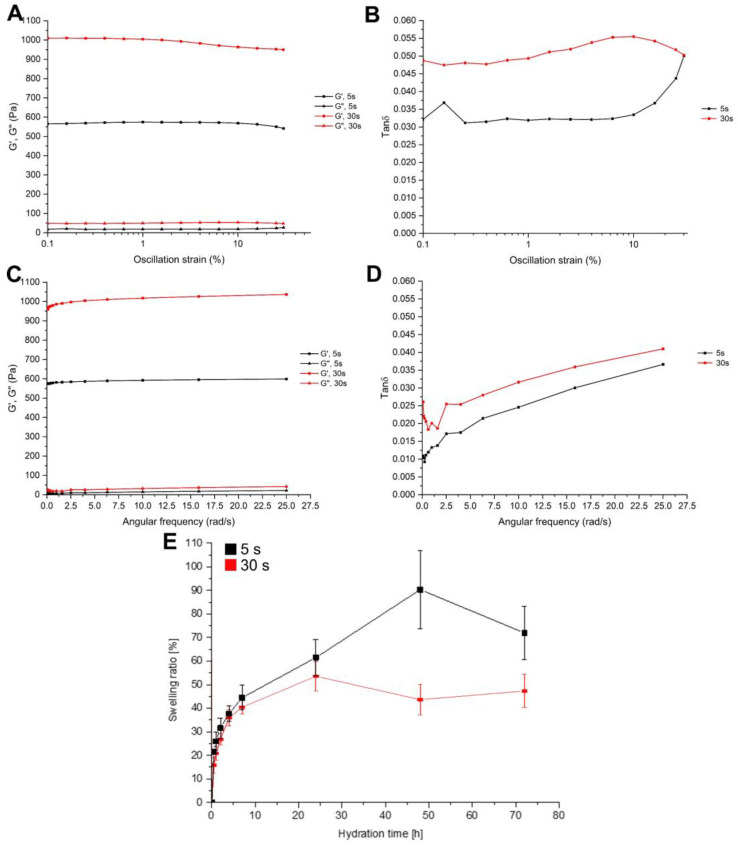
(**A**) Storage (G′) and loss (G″) moduli reported in the function of the oscillation strain (in log scale); all the curve results were linear up to 1%. (**B**) Tan δ in the functioning of the oscillation strain; the trend for the 5 s crosslinked material was different from the other due to the incomplete crosslinking. (**C**) G′ and G″ in the functioning of the angular frequency at a constant strain of 1%. (**D**) Tan δ in the functioning of the angular frequency with a constant strain of 1%. (**E**) Swelling ratio during 72 h with different exposure times (5 s and 30 s); as expected, the less exposed samples tend to sell faster, reaching a higher amount of incorporated water.

The measurements of rheological properties allowed us to understand the changes in mechanical properties with different durations of exposure to UV light. Two curing times were analyzed: 5 s and 30 s. Each sample was analyzed right after photocuring, without adding water, to make comparable measurements. First, an amplitude sweep test was performed to define the linear viscoelastic region (LVR). Indeed, the rheological properties of a viscoelastic material are independent until a certain value of strain. Beyond this critical strain, the material’s behavior is non-linear, and the storage modulus declines. The strain sweep test is shown in Figure 2A, where the storage modulus (G′) and the loss modulus (G″) are plotted as a function of strain rate. G′ and G″ represent the elastic and the viscous part of the hydrogels, respectively. All of the crosslinked hydrogels showed a nearly constant value of G′ and G″ for a wide range of shear strain. Up to 1% shear strain (black line, Figure 2A), all of the samples seem to be in the linear viscoelastic region. This suggests that the samples’ structures were undisturbed in all cases. For this reason, 1% shear strain was fixed for the successive frequency sweep test. Interestingly, tan δ curves (Figure 2B) show different behaviors for the two crosslinking times. While the first, after an initial plateau, increased, the latter, after an initial increase, showed a slight decrease. This can be attributed to the low crosslinking time of the first sample, which did not allow a complete crosslinking.

The structure of hydrogels was further characterized with a frequency sweep test (Figure 2C), where frequency was varied between 0.1 rad/s and 25 rad/s, while the shear rate was kept constant. All of the specimens displayed a behavior nearly independent of the frequency, as is expected from a solid-like material. Indeed, G′ showed much larger dependency, exhibiting a more fluid-like material. In all samples, G′ was larger than G″ by a factor of about 40, which indicates that all of the hydrogels had an elastic behavior even with a low duration of UV exposure. All of the tan δ curves reported in Figure 2D showed the same trend; as expected, the curves were shifted to higher values accordingly with the increase in the exposure time. This indicates a progressively more dissipative material.

The result of the swelling test is shown in Figure 2E: the weight of samples increased with the increasing hydration time (up to 72 h after crosslinking). Nevertheless, the behavior of the two samples was different. The sample cured for 5 s reached a swelling ratio of about 90%, while the sample cured for 30 s limited the swelling to about 45%, also showing a lower swelling rate. As expected, the reduction of the crosslinking degree, determined by the limited curing time, increased the ability of the gel to absorb water, permitting a more pronounced expansion of the polymeric network. Overall, these data showed that the mechanical properties improved with the curing time due to the increase of the crosslinking degree. As a drawback, the ability of the gel to adsorb water decreases. For this reason, to approximately reach the equivalent adsorbed energy of 5 s under the UV lamp (433 W/m^2^), the DLP (45 W/m^2^) exposure time was chosen to be 30 s.

### 2.3. Biological Evaluation

**Figure 3:**
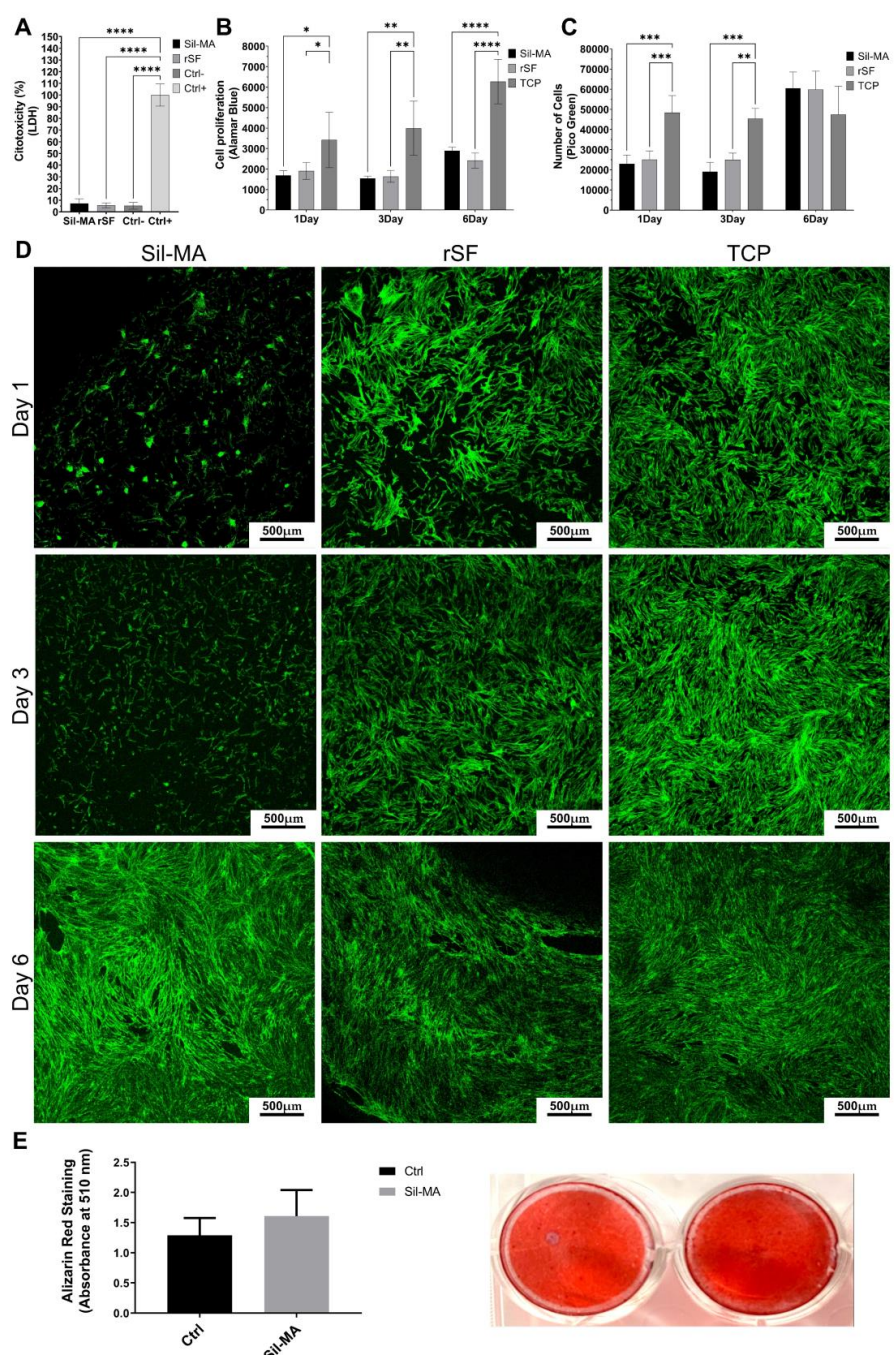
(**A**) Graph of LDH assay; (**B**) graph of Alamar Blue assay; (**C**) graph of Pico Green assay; (**E**) Representative confocal images on days 1, 3, and 6; (**D**) graph representing Alizarin Red staining quantification; representative images of Alizarin Red staining. Groups were considered statistically different with a *p* < 0.05. The confidence levels were assigned as follows: *p* ≤ 0.05 (*), *p* ≤ 0.01 (**), *p* ≤ 0.001 (***), *p* ≤ 0.0001 (****).

A cytotoxicity assay was performed on Sil-MA films to evaluate any possible cytotoxic effect due to the material. This assay is based on the lactate dehydrogenase (LDH) release from damaged cells. rSF films were used as controls. In Figure 3A, the result after 48 h of culture in the medium depleted of phenol red is reported. Sil-MA had a low LDH release, comparable with rSF and the negative control (Ctrl−) represented by cells grown in a plastic dish (TCP). The positive control (Ctrl+) is obtained by lysing the cells to the highest maximum LDH release (100% cytotoxicity). According to these results, Sil-MA films were not cytotoxic.

An Alamar Blue assay evaluated cell metabolic activity on days 1, 3, and 6. The results are shown in Figure 3B. The metabolic activity of Sil-MA films is similar to that of rSF films. The increase in metabolic activity is not so evident from day 1 to day 3, while it increases at day 6. At each time point, the values obtained from cells cultured in adhesion (TCP) are higher than Sil-MA and rSF ones, as expected.

Figure 3C shows the result of a Pico Green assay. The test evaluates cell proliferation, estimating the number of cells at each time point. The number of cells of Sil-MA is similar to that of rSF at each time point; cell proliferation is low at the beginning but markedly increases on day 6. The values seem to be constant on day 1 and day 3, indicating a low proliferation state. The results of Alamar Blue tests supported the evidence for a low proliferation state. The mean number of cells values are reported in detail in Table 1. On day 1 and day 3, both Sil-MA and rSF show approximately the same cell number, while on day 6, it is tripled. The number of cells on TCP remains constant during the test, because the cells reached confluence on day 1, and the proliferation was further inhibited.

In Figure 3D are shown representative images of the confocal analysis of Sil-MA, rSF, and TCP on days 1, 3, and 6. The cytoskeleton is stained with Phalloidin i-Fluor 488, and the nuclei is stained with DAPI. rSF materials absorb DAPI, leading to difficulty identifying the nuclei. For this reason, only cytoskeleton images are shown. Sil-MA samples show low cell adhesion on day 1 and day 3, compared to rSF and TCP. However, on day 6, good cell adhesion on Sil-MA films is reached, comparable to rSF and TCP; cells show a stretched shape and are well-distributed on the film.

To assess whether Sil-MA films can support osteogenesis, human ADSCs were cultured in adherence or on Sil-MA films under osteogenic conditions for 14 days. Figure 3E shows that Sil-MA sustained mineral apposition, as measured by Alizarin Red staining, and induced slightly higher mineralization compared to control samples grown on plastic.

Overall, these that showed that Sil-MA films are not cytotoxic and support cell metabolism, adhesion, proliferation, and osteogenic differentiation.

### 2.4. DLP Fabrication

A variety of structures (grids, multilayered steering wheels, monolayered Biotech logo) were printed on glass slides using the DLP projector to evaluate the printability in terms of resolution, pattern fidelity, and geometric aspect ratio. 

**Figure 4:**
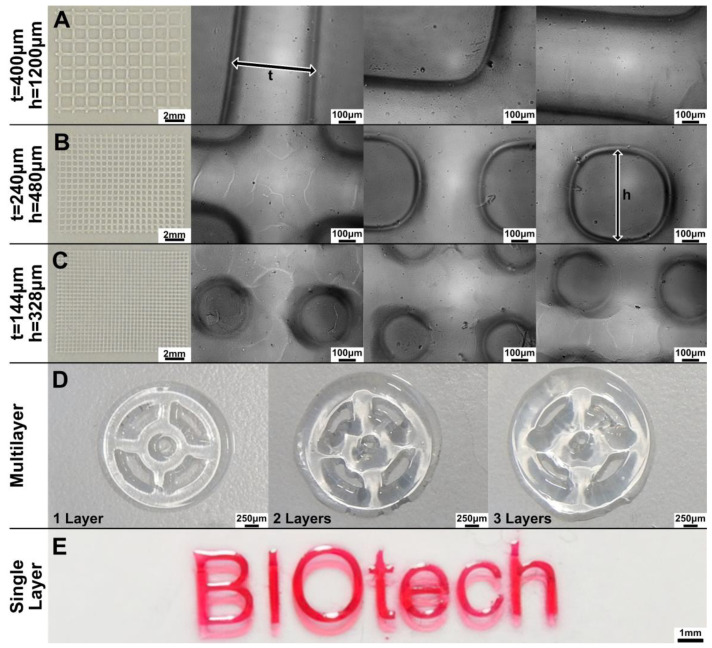
(**A**–**C**) A series of different grids were printed by the DLP projector; t indicates the length of the lines while h the side of the squared pore. By decreasing both t and h, the pores became rounded instead of squared. This could be also due to the imperfect control of the focus in our system. (**D**) Structures with more than one layer (e.g., multilayered steering wheels) were also difficult to print due to the manual adjustment. (**E**) A single-layer structure (e.g., BIOtech logo) was well-defined. The BIOtech logo has been printed, adding red food coloring to enhance the contrast.

The results are shown in Figure 4. The exposure time (60 s per layer) has been chosen considering the results of the previous section. The structures become less defined when finer lines and smaller pores are printed (moving from A to B to C in Figure 4). The theoretical dimension of the projected pixel is 16 μm, as evinced from the DLP resolution (1140 × 912 pixels), and the dimension of the projection is 18 × 15 mm. However, the limited depth of field of the projector’s optics and the tendency of the Sil-MA hydrogel to absorb water during the development steps (which imply several washings in water) did not allow us to print structures close to the nominal resolution. Using the manual focus, adjustment up to three layers was performed and ‘multilayered steering wheels’ constructs printed (Figure 4D); however, the system performs better on a single layer, as in the case of the printed logo, (Figure 4E), since multiple layers underwent double or triple defocused UV exposure. In fact, while the first layer was well-defined, shape definition was lost right at the second printed layer. 

**Figure 5:**
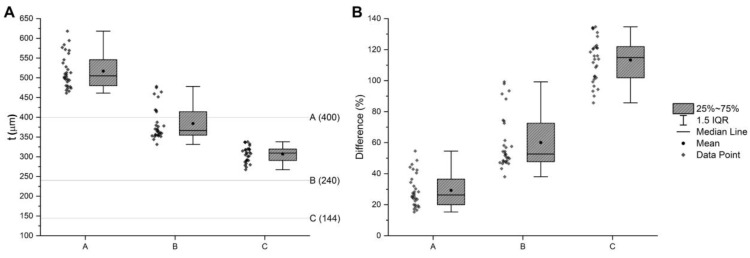
(**A**) Box plot of the line thickness of the first three structures of Figure 2. The lines indicate the nominal quotation. (**B**) Box plot of the percentage difference between the measured and the nominal thickness. The difference should be 0. All of the measures were higher than the nominal value, and more packed structures with a smaller line thickness had a higher percentage difference.

The coherency between the nominal and printed dimensions was estimated in the case of grid structures (Figure 4A–C)and reported in Figure 5 and Table 2. The measured line thickness was always higher than the nominal quotation. Interestingly, with a smaller thickness, the percentage difference with the nominal quotation was higher. The reason for this can be understood by observing Figure 4A–C. In fact, the squared pores become circular, moving from the first to the third layer, making the dividing lines larger.

### 2.5. Pneumatic Extrusion Printing

**Figure 6:**
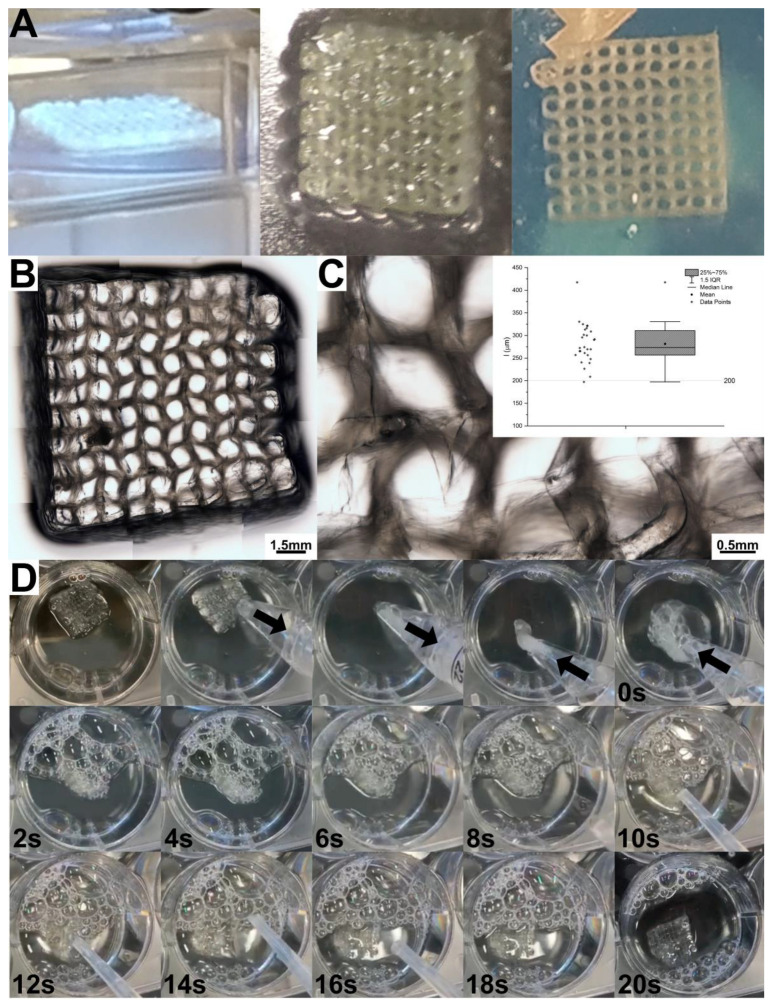
(**A**) Crosslinking of the 3D extrusion-printed Sil-MA structure by UV-irradiation and obtained structure embedded in poloxamer. (**B**) Brightfield microscopy, composed by several micrographies and (**C**) a singular micrography, wherein the quantification of the filament dimension has been performed. (**D**) Withdrawal, ejection, and shape recovery of a Sil-MA 10-layer structure.

Sil-MA was also printed with a bioprinter using a pneumatic extrusion technique in a poloxamer support bath. In this case, a higher concentration of Sil-MA and photoinitiator has been used to allow the correct formation of the filament and the retention of the design dimension. The poloxamer bath was necessary to permit the maintenance of the shape before the UV-crosslinking (Figure 6A) and to print 10-layer structures. After irradiation, the resulting structures were yellowish, as expected from SF solutions, with a concentration above 10%, as previously reported in the literature [34]. A bright field image composed of several micrographs of the printed grid incorporated in pluronic hydrogel is shown in Figure 6B, while a single micrograph is shown in Figure 6C. As expected, the extrusion-printed structure was less defined than the DLP structure. The quantification of the filament dimension (Figure 6C) revealed a mean dimension of 281 ± 41 μm, approximately 40% higher than the nozzle diameter (200 μm). This is an expected phenomenon since, after extrusion, the filament increases its dimension due to stress relaxation [40,41]. The printed structures were proven to retain their shape once ejected from a nozzle. In Figure 6D, we report, as an example, an extrusion-printed 10-layer grid structure after the removal of the supporting poloxamer. The structure has been 3D-printed as detailed above, then withdrawn into a cup pipette (2 mm of diameter), and successively ejected. After 20 s in water, upon rehydration, the grid opened up, recovering its original shape. 

## 3. Discussion

Sil-MA is a methacrylated biopolymer developed to fabricate SF constructs using light-based 3D additive manufacturing. In this work, we developed a prototypical grid structure based on Sil-MA, which showed three main characteristics: it can be folded into a small volume, can be ejected through an insertion needle, and can fully recover its shape after complete rehydration.

The material characterization has revealed that we were able to successfully methacrylate the SF with a rate of functionalization of about 30% (demonstrated by TNBs assay and NMR), which is similar to the rate reported in previous works for the same reaction [27,34]. 

Sil-MA-based hydrogels have been shown in the literature to have good mechanical performances, increasing the interest in the TE applications. In particular, Sil-MA gels were reported having compressive and elastic moduli increasing with Sil-MA concentration, achieving 130 kPA in compression and 15 kPa in tension for gels produced with a 30% Sil-MA solution [27]; these values are close to the elasticity modulus of the normal bone extracellular matrix (ECM), 20–50 kPa [42], and far from the elasticity modulus of the mineralized bone, whose values are as large as 10–20 GPa [43]. Herein, we did not test the Sil-MA constructs by compression given their thickness. Therefore, this construct is not suitable for load-bearing applications but rather for cavities or surface filling. 

Other silk-related materials were developed with the specific purpose of structural applications [11,25]. The rheological test performed gave results of G′ and G″ higher than those reported in literature [39]; this is easily understandable, considering that, even though the starting material was the same, the crosslinking degree after UV-exposure depends on a series of other factors, which vary from experiment to experiment (adsorbed energy, wavelength of the UV lamp, concentration of Sil-MA, and amount of LAP).

Sil-MA has been widely used as bioink and proven to be non-cytotoxic when cells were included in the ink [27,38]. The vitality was reported to be UV-exposure-dependent and to also have a dependency on the amount of added photoinitiator that, above a certain amount, results in cytotoxicity [34]. In addition, when cells were included into the material to perform 3D cell cultures, the increase in the Sil-MA concentration decreased the cell viability [39]. The threshold for the 3D cell culture resulted in a Sil-MA concentration of 10% [39]. In this study, Sil-MA has not been used as bioink, which, by definition, requires cells prior to bioprinting, but as a base material to build implantable construct. For this reason we could perform the 3D printing with a higher Sil-MA concentration (up to 20%) without compromising the cells vitality. The material was tested, for the first time to our knowledge, following the international standards organization protocols (ISO-10993) on the MRC5 cell line to certify its biocompatibility and indicated an absence of cytotoxicity. Moreover, additional in vitro biological evaluation on MRC5 cells lines showed good adhesion, proliferation, and metabolic activity. However, MRC5 in the first days showed low proliferation compared to rSF, which could be partially explained by the possible residual presence of GMA or LiBr, which inhibit the proliferation ability of cells. Previous authors showed that the ideal dialysis period for the modification of SF and GMA is seven days to preserve a good cell proliferation rate [44].

We also performed a test to evaluate the osteoinductivity of Sil-MA using human ADSCs to prove if Sil-MA can support osteogenesis. Our data confirmed that methacrylation does not negatively interfere with osteogenesis and that Sil-MA displays osteoconductive properties similar to those of SF sponges in our previous study [45].

In terms of 3D printing, the DLP projector ensured better-resolved features when compared with pneumatic extrusion. This was expected, considering the higher resolution and precision of the optical system. However, if compared to the literature [27], our DLP-printed structures did not reach the theoretical resolution of the projector. This can be be attributed to the manual focusing and the consequent broadening of the structure when compared to the design. When compared to GelMA printed with a DLP, the resolution obtainable is similar. In fact, GelMA has been recently printed with a resolution of 200 μm by a DLP printer with a maximum resolution of 50 μm [46]. When pneumatic extrusion was used, an increase in the dimension occurred in the Sil-MA structures and should be attributed to the flow stresses relaxation, a phenomenon widely discussed in the literature [40,41].

Other authors previously showed memory-shape features of 3D-printed scaffolds made of fibroin. As an example, a memory-shape-implant meniscus was produced by an enzymatic cross-link of rSF with horseradish peroxidase [47], but in this case, the produced structure was not injectable. An injectable, photoluminescent, carbon-nanotubes-doped sericin scaffold (CNTs-SS) with a programmable shape-memory property was developed for stroke applications, showing how this type of scaffolds can be readily compressed into small volumetric mass (roughly 90%) and conveniently injected into stroke cavities, followed by shape restoration triggered by fluids [48]. However, to our knowledge the possibility of injecting a SF-structured scaffold was not reported before.

While promising, our study has several limitations. First, we failed to perform multilayer structures in case of DLP because of the loss of resolution; further studies need to be performed to tune this aspect. In case of pneumatic extrusion, we reached the printing of 10 layers, thanks to the poloxamer support bath, but also in this case with an important loss in terms of resolution. 

Further studies should be performed on the 3D-printed constructs to evaluate cell seeding, adhesion, proliferation, and differentiation in vitro, as well as neo tissue formation and biodegradability in vivo. Whether these shape-memory properties would be maintained in vivo has yet to be confirmed: further in vivo analysis should be performed to successfully prove the possibility of inserting the scaffold into a bone cavity or surface and verify the effectiveness in recovering its shape to fit the whole space and the capacity to promote bone formation.

## 4. Conclusions

In this work, we proved the possibility of printing a Sil-MA thin layered structure able to recover its shape after being ejected. Both the use of a DLP projector and a pneumatic extrusion were suitable for the production of thin structures. In both cases, the dimension of the crosslinked Sil-MA was larger than the design quotation. However, the resulting structures were acceptable. An in vitro biological assessment of the biocompatibility has been conducted following the ISO protocols (ISO 10993). Sil-MA results were biocompatible. An additional biological test proved the ability of Sil-MA to support osteogenesis. Finally, a construct has been withdrawn and ejected through a small insertion needle, proving its ability to recover its shape. Further studies will be necessary in the future to verify in depth the use of Sil-MA in case of bone regeneration in terms of biological performances. 

## 5. Materials and Methods

### 5.1. Sil-MA Preparation

**Figure 7:**
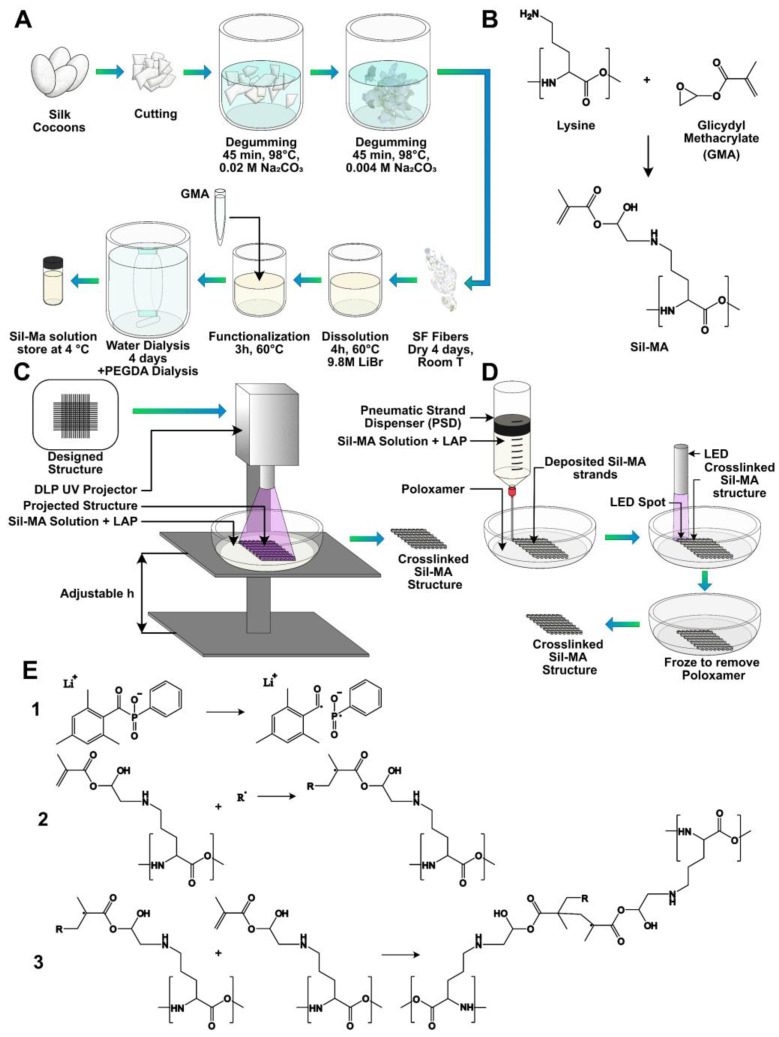
(**A**) Scheme of Sil-MA production starting from Bombyx Mori silk cocoons and (**B**) supposed chemical reaction for the formation of the Sil-MA; a primary amide in the lysine group became a di-β-hydroxyamide, through the nucleophilic substitution of nitrogen on one carbon atom of the epoxy ring of GMA and the consequent ring opening. (**C**) Scheme of the Sil-MA constructs formation through the digital light processing (DPL) projector. (**D**) Scheme of the printing through the use of a pneumatic strand dispenser (PSD) inside a poloxamer support bath, of the crosslinking with LAP and of the removal of the poloxamer by decreasing the temperature below the gel-to-sol transition. (**E**) Scheme of the crosslinking reaction. (1) Formation of radicals under UV light because of the photoinitiator, LAP. (2) Formation of radicals on the protein chain because of the effect of the radicals formed by the photoinitiator (here indicated with R) and the opening of the carbon double bond. (3) Formation of a cross-linking between two chains because of the opening of the carbon double bond.

Bombyx mori silkworm cocoons were obtained from Chul Thai Silk Co., Phetchaban, Thailand, and degummed for extracting SF, following a previously published protocol briefly illustrated in Figure 7A [12]. Each silkworm cocoon was cut into two pieces and delaminated into two foils. A unit of 30 g of delaminated cocoon was first boiled for 45 min at 98 °C in a boiling bath containing an aqueous solution of 0.02 M Na_2_CO_3_ (3.3 g of sodium carbonate, Sigma-Aldrich, in 3 l of distilled water) and then boiled for other 45 min at 98 °C in a boiling bath containing an aqueous solution of 0.004 M Na_2_CO_3_ (1.2 g of sodium carbonate in 3 l of distilled water). For completely removing sericin and the residual salt, SF was rinsed out in distilled water, lowering the bath temperature step by step, in order to not expose SF to high temperature gradients. When room temperature was reached, SF was further washed in distilled water. The degummed SF was dried under the hood at room temperature for 48 h. Degummed SF was dissolved in a 9.3 M LiBr (Honeywell Fluka, Cat. 746479) previously prepared, at 20% *w*/*v* concentration for 3 h at 65 °C in the oven. SF must be completely dissolved and appear clear after 3 h.

Successively, glycidyl methacrylate (GMA, Sigma-Aldrich, 151238) was added in the beaker in this proportion: 1 mL of GMA per 4 g of SF, chosen according to Soon Hee Kim et al. [27]. The reaction was carried out, stirring with 300 rpm at 60 °C for 3 h. The hypothesized chemical reaction of the modification is reported in Figure 7B: a di-β-hydroxyamide group is supposed to form from the primary amine (of the lysine side group) in SF and the epoxy ring of GMA, through the nucleophilic substitution of nitrogen on one carbon atom of the epoxy ring and the consequent ring opening. After reaction, the solution appeared cloudy. The mixture obtained was dialyzed against distilled water using a 3.5 kDa cutoff dialysis membrane for 4 days, in order to remove excess salt and GMA, which is a toxic reagent. At the end, the concentration was measured by UV spectroscopy (Nanordop 1000, ThermoFisher Scientific, Waltham, MA, USA), and the solution was dialyzed again against a 25% *w*/*v* concentrated PEGDA solution, using a 3.5 kDa cutoff dialysis cassette to concentrate Sil-MA. The dialysis was stopped when the solution concentration of Sil-MA reached 10% *w*/*v*.

### 5.2. Infrared Spetroscopy

FTIR spectroscopy was used to qualitatively confirm the presence of methacrylic groups in the Sil-MA structure. A FTIR spectrometer (Spectrum ONE, PerkinElmer, Waltham, MA, USA) was used, and the samples were analyzed in UATR (universal attenuated total reflectance) mode. The wavenumber range analyzed was 4000–650 cm^−1^. The spectra were acquired as a mean of 16 scans with a resolution of 1 cm^−1^.

### 5.3. UV Specrtoscopy

A quantitative analysis was done by means of a 2,4,6-trinitrobenzene sulfonic acid (TNBS) assay, a sensitive assay reagent for the determination of free amino groups, in order to quantify the methacrylation degree in Sil-MATNBS; upon reaction with primary amines, it forms a highly chromogenic product whose absorbance at 335 nm to 345 nm can be measured. As GMA reacts with free amino groups in SF, which are mostly present in lysine, the calculation of the remaining free amino groups in Sil-MA can be used to identify the methacrylation degree. TNBS assay was performed on SF and Sil-MA to quantify the difference in free amino groups.

TNBS (Sigma-Aldrich, 92822) was diluted in a 0.1 M sodium bicarbonate buffer (pH = 8.5). The stock solutions for the samples were prepared at a concentration of 2.43 mg/mL, measured by NanoDrop. Then, 100 µL of each sample stock solutions were incubated at 37 °C for 2 h in 100 µL of TNBS solution. To stop and stabilize the reaction, 250 µL of 10% *w*/*v* SDS and 125 µL of 1 M HCl were added to each sample after incubation. The calibration curve was generated by the use of several standard samples composed of water solutions containing increasing concentrations of beta alanine (PHR1349, Sigma Aldrich, St. Louis, MO, USA), which is known to have one amino group. The used concentrations are reported in Table 3. All of the absorbance measurements were taken using a microplate reader (Spark 10M, Tecan, Hombrechtikon, Switzerland), at 340 nm. All of the absorbance measurements were subtracted from the value of the blank (consisting of all the reagents used, without beta alanine).

The analysis is based on the Lambert–Beer law (Equation (2)). The calibration curve can be generally represented by Equation (3), and, by inverting it (Equation (4)), we were able to calculate the concentration of aminoacidic groups in the bare SF (C_rSF_), the concentration of the aminoacidic group in the modified Sil-MA (C_Sil-MA_), and the percentage of substitution (DS, Equation (5)).

(2) A=εlC

(3) $$A=mC+A_{0}$$

(4) $$C=\frac{A-A_{0}}{m}$$

(5) $$DS\left(\%\right)=\frac{C_{rSF}-C_{Sil-MA}}{C_{rSF}}*100$$

### 5.4. Nuclear Magnetic Resonance (NMR)

In order to confirm the result of quantitative optical spectroscopy, SF and sil-MA were examined by 1H liquid NMR using a Bruker (Billerica, MA, USA) Ultrashield Plus spectrometer (9.4 T) at a frequency of 400 MHz. A total of 100 mg of each sample was dissolved in 500 µL of a 9.3 M LiBr solution. Then they were diluted in D_2_O. The degree of methacrylation was defined according to the lysine groups in SF that are modified in Sil-MA. The spectra were analyzed removing the signal of deuterium, and the signal at 1 ppm was used to normalize each spectrum. Then the lysine signal (2.9 ppm) of the two samples was integrated. The substitution degree was calculated following Equation (6).

(6) $$DS\left(\%\right)=1-\frac{lysine\;integration\;signal\;on\;Sil-MA}{lysine\;integration\;signal\;on\;rSF}\cdot 100$$

### 5.5. Water Uptake

A total of 500 µL of Sil-MA were cured following the crosslinking procedure explained above. Two curing times were considered: 5 s and 30 s. Furthermore, 5 mL vials made of polystyrene transparent to UV light were used. The samples were weighed just after crosslinking to obtain *W_cross_*. Then they were hydrated for 0.5, 1, 2, 4, 7, 24, 48, and 72 h in distilled water and maintained in a refrigerator during the test. Water was removed at each sampling time, and hydrogels were weighed to obtain *W_swallen_*. The water uptake was calculated according to Equation (7).

(7) $$SW\;\left(\%\right)=\frac{\left(W_{swallen}-W_{cross}\right)}{W_{cross}}\cdot 100$$

### 5.6. Rheology

Rheological measurements were performed to understand how the mechanical properties change with changing curing time. The rheological properties of Sil-MA hydrogels were measured using a stress control rheometer (Discovery HR-2, TA Instruments) equipped with a Peltier plate for temperature control. The measurements were performed using parallel plate geometry (40 mm in diameter) at 25 °C. Disc-shaped samples 40 mm in diameter and 2 mm thickness were crosslinked using a cylindrical mold and a UV LED lamp. Three samples with different curing times were analyzed: 5 s and 30 s. The storage modulus (G′) and loss modulus (G″) were measured in oscillatory mode. Firstly, all of the samples underwent a strain sweep test in order to find the linear viscoelastic region, then frequency sweep tests were performed inside this region. In the strain sweep measurements, the samples were tested, keeping constant angular frequency at 10 rad/s and varying the strain from 0.1 % to 30%, acquiring five points per decade in logarithmic scale. For frequency sweep measurements, the samples were tested at varying angular frequency from 0.1 to 25 rad/s (logarithmic scale, 5 points pe decade), keeping shear strain constant at 1%. One sample cured for 5 s was also tested from 0.1 to 100 rad/s. All of the tests were carried out under axial force control (2 N) during gap closure.

### 5.7. Morphology

The morphology of the construct was evaluated by optical microscopy (Eclipse 90i, Nikon, USA) comparing the printed object with the design to evaluate its consistency. The images were analyzed by FIJI (v. 1.53t, National Institute of Health, Bethesda, MD, USA).

### 5.8. In-Vitro Biological Evaluation

Sil-MA and rSF were tested in the form of film, both prepared with the same methodology. The lyophilized rSF was dissolved in formic acid at 8% *w*/*v* concentration. Sil-MA films were produced starting from a sil-MA water solution with a 10% w/v concentration and adding 0.1% *w*/*v* of LAP photoinitiator. The obtained mixtures were poured and cast in a Petri dish overnight. When all of the formic acid was evaporated, rSF and Sil-MA films were exposed to UV light for 30 s to stabilize them. All films were washed twice in distilled water to eliminate possible formic acid residues. rSF and Sil-MA were sterilized using 70% ethanol for 30 min at room temperature before being placed on 48-well plates.

Biological tests were performed on Sil-MA and rSF to verify the potential use of this material in TE. A control of cells cultured in adhesion was also used (ctrl−, TCP). The effects of Sil-MA were evaluated on cytotoxicity, cell metabolism, and cell adhesion using the human lung fibroblast (MRC5) cell line, in compliance with the European Standard EN ISO 10993-12:2004 and 10993-5:2009.

MRC5 were expanded upon reaching 70% confluence. Then they were detached and seeded at 15 × 10^3^ cells/cm^2^ into 48-well plates containing Sil-MA substrates. 

#### 5.8.1. Lactate Dehydrogenase (LDH) Assay

Cytotoxicity was evaluated by means of an LDH assay (ThermoFisher Scientific, Waltham, MA, USA), following the manufacturer’s instructions. Four experimental groups were tested, as described in Table 4. The MRC5 cell line as seeded in a 96-well tissue culture plate at a concentration of 5000 cells/well and cultured in standard medium until about 70% confluence. Then the medium was replaced with 100 µL/well of phenol red and with heat-inactivated serum. The volume of the medium was calculated according to ISO-10993-12:2004 (small, molded items, thickness: 0.5–1 mm, extraction ratio area/volume: 3 cm^2^/mL). The surnatant was harvested at 48 h of culture and analyzed. For each group, five technical replicates were performed. The colorimetric detection of LDH was performed at 492–620 nm on a spectrophotometer, and cytotoxicity was calculated as a fold decrease compared to the positive control (100% of cytotoxicity).

#### 5.8.2. Alamar Blue Assay

Metabolic activity was evaluated by means of an Alamar Blue assay. MRC5 cells line were seeded on the different experimental groups (Sil-MA films, rSF films, on adhesion-TCP). After 1, 3, and 6 days of culture, the medium was discarded, and the samples were incubated with 1 mL of medium containing 10% Alamar Blue reagent (Resazurin, R7017, Sigma Aldrich, St. Louis, MO, USA) for 1.5 h at 37 °C. Then, 100 µL of the supernatant were transferred to a black 96-well plate; technical duplicates were performed for each sample. A negative control, 100 µL of medium containing 10% Alamar Blue reagent, was also analyzed. The fluorescence intensity was measured using a microplate reader (Spark 10M, Tecan): the excitation wavelength was 590 nm, while the emission wavelength was 535 nm.

After the analysis, the seeded films were washed twice with PBS and successively used to perform the Pico Green assay.

#### 5.8.3. Pico Green Assay

Cell adhesion and proliferation was evaluated by using a Pico Green fluorescence assay kit (Pico Green assay, P11496, Invitrogen, Waltham, MA, USA) according to the manufacturer’s instructions, which quantifies the DNA in biological samples and thus the cell number. At each time point (day 1, day 3, day 6), 250 µL of 0.05% Triton-X were poured in each well, and after 20 min, the well plate was moved to a −20 °C refrigerator until the test was carried out. The procedure needed a calibration step, in which samples with known DNA concentration were used in order to build a calibration curve. A solution of the Tris-EDTA buffer was prepared, diluting the Tris-EDTA buffer 20 times provided by the kit with distilled water. A DNA working solution was prepared by adding 20 µL of the stock solution (100 µg/mL) in 980 µL of the Tris-EDTA buffer. The samples for the standard curve were prepared using different composition of the Tris-EDTA buffer and DNA working solution (Table 5).

Each sample was sonicated for 10 s and diluted 1:1 with the Tris-EDTA buffer to obtain 500 µL of final volume. The Pico Green working solution was prepared by diluting 50 µL of Pico Green stock solution in 10 mL of Tris-EDTA buffer (200-fold dilution). A total of 100 µL of all the samples was moved to a black 96-well plate in triplicates. A total of 100 µL of the Pico Green working solution as added to each well and incubated at room temperature for 2–3 min. The fluorescence intensity was measured using a microplate reader (Spark 10M, Tecan): the excitation wavelength was 485 nm, while the emission wavelength was 535 nm.

### 5.9. Confocal Microscopy

Confocal analysis (Nikon A1 Confocal Microscope, ) was performed in order to estimate cell adhesion on Sil-MA films, compared to rSF and cells cultured in adhesion. At day 1, 3, and 6, cells were fixed with 4% paraformaldehyde (PFA) at room T for 20 min. Successively, cell membrane was permeabilized using 0.2 % Triton X-100 solution, and nuclei were stained with DAPI (5.4 µL in 25 mL PBS), whereas the cytoskeleton actin was stained with Phalloidin i-Fluor 488 (5 µL in 25 mL PBS) (ab176753, Abcam, Cambridge, UK). After staining, the samples were washed three times with PBS and stored at 4 °C.

### 5.10. Osteogenesis

To investigate whether the constructs could play a role in supporting osteogenic differentiation, human ADSCs (Lonza, Basel, Switzerland) were seeded onto the Sil-MA films and rSF films or in adhesion and cultured under osteogenic condition for 14 days in α-MEM 20% FBS supplemented with 100 nM dexamethasone, 100 µM ascorbic acid, and 10 mM β-glycerophosphate. At Day 14, Alizarin Red S (AR-S) (Sigma Aldrich, St. Louis, MO, USA) staining was performed to assess the presence and extent of mineralization. Briefly, cells were stained with 40 mM AR-S for 20 min after being fixed for 15 min at RT in formaldehyde (Kaltek, Padova, Italy) in 10% phosphate buffered saline (PBS) and washed twice with PBS. A spectrophotometric analysis with a TECAN Infinite^®^ 200 PRO (Tecan Italia S.r.l., Cernusco Sul Naviglio, Italy) was performed to quantify the mineral apposition.

### 5.11. UV Crosslinking

A water solution of LAP photoinitiator at 1% *w*/*v* concentration was prepared. LAP was added to the Sil-MA solution at 0.1% *w*/*v* concentration. The obtained mixture was exposed to 365 nm UV light using a UV LED lamp (Spot LED 15 W, Unionprint, Milan, Italy). The crosslinking proposed mechanism is shown in Figure 7E. The lamp was placed at a distance of 5 cm from the support plane. In this position, an irradiated spot with a 7 cm diameter was considered. The intensity at 5 cm from the lamp was about 433 W/m^2^ (it was measured with D0971 quantum photo-radiometer and thermometer data-logger by Delta Ohm using the probe for wavelengths of 315–400 nm).

### 5.12. DLP Printing

Structures were developed using a digital light processing (DLP) UV projector (PRO4500 DLP, Wintech, San Marcos, CA, USA) mounted on a custom-made setup. The set-up used is shown in Figure 7C. The sample was poured in a Petri dish fixed to the support plane, and the focus was regulated by means of a screw moving the support plane along the vertical axis. The intensity of the light at 4 cm from the source was about 45 W/m^2^ (measured with a D0971 quantum photo-radiometer and a thermometer data-logger by Delta Ohm, using the probe for wavelengths of 315–400 nm). The emission peak of the projector LED was at 370 nm, and the resolution of the projected image was 1140 × 912 pixels.

After adding LAP to the Sil-MA solution at 0.1% *w*/*v* concentration, 500 µL of the obtained mixture was poured into a Petri dish with 30 mm in diameter and exposed to UV light for 60 s. Patterns were developed by pipetting distilled water on the sample, which allowed uncrosslinked fibroin removal. 

Some trials in fabricating 3D constructs were done using a layer-by-layer approach: after a first layer was crosslinked, the procedure was repeated, adding another 500 µL of solution in the Petri dish and lowering the support plane in order to adjust the focus. The time of UV exposure was 60 s for each layer. The development was done after the crosslinking of the last layer.

### 5.13. Pneumatic Extrusion Printing

The 3D-printing process was also performed by means of a 3D Discovery platform (RegenHU, Villaz-St-Pierre, Switzerland). The procedure is briefly schematized in Figure 7D. First, the structures were designed by means of dedicated CAD software (BioCAD, RegenHU, Villaz-St-Pierre, Switzerland). The scaffolds were realized with a 10 × 10 mm square base and a total height of 2 mm. A 0/90° infill pattern was selected as the internal microarchitecture. The fiber diameter was set to 200 µm and pore size to 800 µm. A layer height of 200 µm was chosen, leading to the stacking of 10 layers to achieve the desired construct height. The design software enabled us to set the printing process to be automatically replicated within six-well plates. The wells were filled with a support gel to retain the ink fibers’ shape until crosslinking was applied, so as to improve the fabricated constructs’ fidelity to the design. 

The support gel was formulated by dissolving Poloxamer 407 (Sigma-Aldrich St Louis, MO, USA) in distilled water, in a 30% *w*/*v* ratio. The gel was formed by gradually adding the polymer to the solvent under magnetic stirring at a temperature of 4 °C to facilitate dissolution. Subsequently, the plate wells were filled with the gel and kept at RT for a minimum of 30 min, until gelation was observed, prior to the printing process.

To fabricate the 3D constructs, the Sil-MA cartridge was loaded in a pneumatic extrusion printhead of the 3D discovery platform. A 200 µm nozzle with a length of 12.7 mm was used. The process was performed at room temperature (RT), with a pressure of 0.6 bar and a printing speed of 10 mm/s. After fabrication, each scaffold was irradiated with a 365 nm UV lamp available in the 3D discovery platform. The photocrosslinking duration was set to 5 min.

Finally, the six-well plates were kept at 4 °C for a time of at least 30 min, until sol transition was observed. The 3D scaffolds were then removed from the wells and rinsed with PBS three times (5 min per cycle) to remove poloxamer support gel residuals.

### 5.14. Characterization of Shape-Memory Property

A proof of principle of the shape-memory property was performed by the immersion of the sil-MA construct in PBS for 30 min. Afterwards, the structure was compressed by aspiration in a pipette cap with a diameter of 2 mm. Then, the construct was extruded from the cap and rehydrated by the addition of PBS until achieving complete shape recovery. 

### 5.15. Statitical Tests

The statistical tests were conducted using R (v. 4.1.3, AT&T, Dallas, TX, USA) in R studio (v. 1.4.1106, Posit, Boston, MA, USA) as IDE. An ANOVA test with a post-hoc Tukey test was performed to verify if there was a statistical difference among the groups. Groups were considered statistically different with a *p* < 0.05. The confidence levels were assigned as follows: *p* ≤ 0.05 (*), *p* ≤ 0.01 (**), *p* ≤ 0.001 (***).

## Figures and Tables

**Table 1:** Number of cells values obtained from the Pico Green test.

	Day 1 [No. Cells]	Day 3 [No. Cells]	Day 6 [No. Cells]
SFMA	22,932	19,005	60,523
SF	25,099	24,990	59,831
TCP	48,429	45,367	47,425

**Table 2:** Descriptive statistic on both the measured line length of structures A, B, and C and their percentage difference with the nominal quotation. In all cases, the lines were thicker than the nominal value. In all cases, the measured thickness was higher than the nominal dimension. Interestingly, more packed structures with a smaller line thickness had a higher percentage difference.

**Line Thickness (μm)**									
**Structure**	**Nominal**	**Mean**	**StD**	**Min**	**Q1**	**Median**	**Q3**	**Max**	**IQR**
A	400	517.05	42.63	461.23	480.08	505.10	545.81	618.28	65.72
B	240	384.20	42.32	331.25	354.70	366.43	414.07	478.13	59.37
C	144	307.14	20.36	267.38	290.68	309.46	319.51	338.01	28.83
**Percentage Difference (%)**									
**Structure**	**Nominal**	**Mean**	**StD**	**Min**	**Q1**	**Median**	**Q3**	**Max**	**IQR**
A	0	29.26	10.66	15.31	20.02	26.27	36.45	54.57	16.43
B	0	60.08	17.63	38.02	47.79	52.68	72.53	99.22	24.74
C	0	113.29	14.14	85.68	101.86	114.90	121.88	134.73	20.02

**Table 3:** Beta alanine concentrations used to build the experimental calibration curve.

Concentration [µg/mL]	Molar Concentration [M]	Absorbance
0	0	0
1.95	2.19 × 10^−5^	0.0115
3.91	4.39 × 10^−5^	0.0299
7.83	8.78 × 10^−5^	0.0547
15.65	1.76 × 10^−4^	0.1185
31.25	3.51 × 10^−4^	0.2056

**Table 4:** Groups used in the LDH assay.

Group	Name	Description
1	Sil-MA	MRC5 cells cultured on Sil-MA films
2	rSF	MRC5 cells cultured on rSF films
3	Ctrl+	MRC5 cells cultured on adhesion and then lysed to obtain the maximum level of cytotoxicity
4	Ctrl-	MRC5 cells cultured on adhesion (CTRL−, TCP)

**Table 5:** Concentrations of DNA used to build the calibration curve.

DNA Working Sol. 2 µg/mL (µL)	TE Buffer (µL)	Final Concentration (ng/mL)
400	0	2000
200	200	1000
100	300	500
40	360	200
20	380	100
10	390	50
4	396	20
0	400	0

## Data Availability

Data is available from the authors upon reasonable request.

## Abbreviations

3D	three dimensional
ADSCs	adipose-derived stem cells
ATR	Attenuated Total Reflectance
BTE	Bone tissue engineering
CAD	Computer Aided Design
DLP	Digital Light Processing
DS	degree of substitution
ECM	Extracellular matrix
FTIR	Fourier Transform Infrared Spectroscopy
GMA	Glycidyl Methacrylate
ISO	International Standardization Organization
LAP	lithium phenyl-2,4,6-trimethylbenzoylphosphinate
LDH	lactate dehydrogenase
LDH	lactate dehydrogenase
LVR	linear viscoelastic region
MRC5	human lung fibroblast cell line
NMR	Nuclear Magnetic Resonance
PBS	Phosphinate Buffered Saline
PSD	pneumatic strand dispenser
rSF	regenerated Silk Fibroin
SF	silk fibroin
Sil-MA	Methacrylated Silk Fibroin
SLA	stereolithography
TCP	Tissue Culture Plate
TE	Tissue engineering
Tris-EDTA	Tris-Ethylenediaminetetraacetic acid
TNBS	2,4,6-Trinitrobenzene Sulfonic Acid
TNBS	2,4,6-Trinitrobenzene Sulfonic Acid
UATR	universal attenuated total reflectance
UV-Vis	Ultraviolet Visible

## References

[1] Ahmed E.M. (2015). Hydrogel: Preparation, characterization, and applications: A review. J. Adv. Res..

[2] Moysidou C.M., Barberio C., Owens R.M. (2021). Advances in Engineering Human Tissue Models. Front. Bioeng. Biotechnol..

[3] Chai Q., Jiao Y., Yu X. (2017). Hydrogels for Biomedical Applications: Their Characteristics and the Mechanisms behind Them. Gels.

[4] Díaz A., Puiggalí J. (2017). Hydrogels for Biomedical Applications: Cellulose, Chitosan, and Protein/Peptide Derivatives. Gels.

[5] Bai X., Gao M., Syed S., Zhuang J., Xu X., Zhang X.-Q. (2018). Bioactive hydrogels for bone regeneration. Bioact. Mater..

[6] Fu J., Yang F., Guo Z. (2018). The chitosan hydrogels: From structure to function. New J. Chem..

[7] Yang Y.J., Ganbat D., Aramwit P., Bucciarelli A., Chen J., Migliaresi C., Motta A. (2019). Processing keratin from camel hair and cashmere with ionic liquids. Express Polym. Lett..

[8] Augst A.D., Kong H.J., Mooney D.J. (2006). Alginate Hydrogels as Biomaterials. Macromol. Biosci..

[9] López-Marcial G.R., Zeng A.Y., Osuna C., Dennis J., García J.M., O’Connell G.D. (2018). Agarose-Based Hydrogels as Suitable Bioprinting Materials for Tissue Engineering. ACS Biomater. Sci. Eng..

[10] Milcovich G., Antunes F.E., Farra R., Grassi G., Grassi M., Asaro F. (2017). Modulating carbohydrate-based hydrogels as viscoelastic lubricant substitute for articular cartilages. Int. J. Biol. Macromol..

[11] Bucciarelli A., Chiera S., Quaranta A., Yadavalli V.K., Motta A., Maniglio D. (2019). A Thermal-Reflow-Based Low-Temperature, High-Pressure Sintering of Lyophilized Silk Fibroin for the Fast Fabrication of Biosubstrates. Adv. Funct. Mater..

[12] Bucciarelli A., Greco G., Corridori I., Pugno N.M., Motta A. (2021). A Design of Experiment Rational Optimization of the Degumming Process and Its Impact on the Silk Fibroin Properties. ACS Biomater. Sci. Eng..

[13] Bucciarelli A., Greco G., Corridori I., Motta A., Pugno N.M. (2021). Tidy dataset of the experimental design of the optimization of the alkali degumming process of Bombyx mori silk. Data Br..

[14] Yang Y., Chen X., Ding F., Zhang P., Liu J., Gu X. (2007). Biocompatibility evaluation of silk fibroin with peripheral nerve tissues and cells in vitro. Biomaterials.

[15] Vepari C., Kaplan D.L. (2007). Silk as a biomaterial. Prog. Polym. Sci..

[16] Gupta P., Lorentz K.L., Haskett D.G., Cunnane E.M., Ramaswamy A.K., Weinbaum J.S., Vorp D.A., Mandal B.B. (2020). Bioresorbable silk grafts for small diameter vascular tissue engineering applications: In vitro and in vivo functional analysis. Acta Biomater..

[17] Cao Y., Wang B. (2009). Biodegradation of Silk Biomaterials. Int. J. Mol. Sci..

[18] Bucciarelli A., Mulloni V., Maniglio D., Pal R.K., Yadavalli V.K., Motta A., Quaranta A. (2018). A comparative study of the refractive index of silk protein thin films towards biomaterial based optical devices. Opt. Mater..

[19] Parker S.T., Domachuk P., Amsden J., Bressner J., Lewis J.A., Kaplan D.L., Omenetto F.C. (2009). Biocompatible silk printed optical waveguides. Adv. Mater..

[20] Perotto G., Zhang Y., Naskar D., Patel N., Kaplan D.L., Kundu S.C., Omenetto F.G. (2017). The optical properties of regenerated silk fibroin films obtained from different sources. Appl. Phys. Lett..

[21] Bucciarelli A., Motta A. (2022). Use of Bombyx mori silk fibroin in tissue engineering: From cocoons to medical devices, challenges, and future perspectives. Biomater. Adv..

[22] Whittaker J.L., Choudhury N.R., Dutta N.K., Zannettino A. (2014). Facile and rapid ruthenium mediated photo-crosslinking of Bombyx mori silk fibroin. J. Mater. Chem. B.

[23] Applegate M.B., Partlow B.P., Coburn J., Marelli B., Pirie C., Pineda R., Kaplan D.L., Omenetto F.G. (2016). Photocrosslinking of Silk Fibroin Using Riboflavin for Ocular Prostheses. Adv. Mater..

[24] Zhou B., Wang P., Cui L., Yu Y., Deng C., Wang Q., Fan X. (2017). Self-Crosslinking of Silk Fibroin Using H2O2-Horseradish Peroxidase System and the Characteristics of the Resulting Fibroin Membranes. Appl. Biochem. Biotechnol..

[25] Bucciarelli A., Janigro V., Yang Y., Fredi G., Pegoretti A., Motta A., Maniglio D. (2021). A genipin crosslinked silk fibroin monolith by compression molding with recovering mechanical properties in physiological conditions. Cell Reports Phys. Sci..

[26] Zhang K., Qian Y., Wang H., Fan L., Huang C., Yin A., Mo X. (2010). Genipin-crosslinked silk fibroin/hydroxybutyl chitosan nanofibrous scaffolds for tissue-engineering application. J. Biomed. Mater. Res.-Part A.

[27] Kim S.H., Yeon Y.K., Lee J.M., Chao J.R., Lee Y.J., Seo Y.B., Sultan M.T., Lee O.J., Lee J.S., Yoon S. (2018). Precisely printable and biocompatible silk fibroin bioink for digital light processing 3D printing. Nat. Commun..

[28] Li W., Mille L.S., Robledo J.A., Uribe T., Huerta V., Zhang Y.S. (2020). Recent Advances in Formulating and Processing Biomaterial Inks for Vat Polymerization-Based 3D Printing. Adv. Healthc. Mater..

[29] Bucciarelli A., Pal R.K., Maniglio D., Quaranta A., Mulloni V., Motta A., Yadavalli V.K. (2017). Fabrication of Nanoscale Patternable Films of Silk Fibroin Using Benign Solvents. Macromol. Mater. Eng..

[30] Kurland N.E., Dey T., Kundu S.C., Yadavalli V.K. (2013). Precise patterning of silk microstructures using photolithography. Adv. Mater..

[31] Liu W., Zhou Z., Zhang S., Shi Z., Tabarini J., Lee W., Zhang Y., Gilbert Corder S.N., Li X., Dong F. (2017). Precise Protein Photolithography (P3): High Performance Biopatterning Using Silk Fibroin Light Chain as the Resist. Adv. Sci..

[32] Hong H., Seo Y.B., Kim D.Y., Lee J.S., Lee Y.J., Lee H., Ajiteru O., Sultan M.T., Lee O.J., Kim S.H. (2020). Digital light processing 3D printed silk fibroin hydrogel for cartilage tissue engineering. Biomaterials.

[33] Kim S.H., Hong H., Ajiteru O., Sultan M.T., Lee Y.J., Lee J.S.J.S., Lee O.J., Lee H., Park H.S., Choi K.Y. (2021). 3D bioprinted silk fibroin hydrogels for tissue engineering. Nat. Protoc..

[34] Bucciarelli A., Muthukumar T., Kim J.S., Kim W.K., Quaranta A., Maniglio D., Khang G., Motta A. (2019). Preparation and Statistical Characterization of Tunable Porous Sponge Scaffolds using UV Cross-linking of Methacrylate-Modified Silk Fibroin. ACS Biomater. Sci. Eng..

[35] Wu X., Zhou M., Jiang F., Yin S., Lin S., Yang G., Lu Y., Zhang W., Jiang X. (2021). Marginal sealing around integral bilayer scaffolds for repairing osteochondral defects based on photocurable silk hydrogels. Bioact. Mater..

[36] Mao Z., Bi X., Wu C., Zheng Y., Shu X., Wu S., Guan J., Ritchie R.O. (2022). A Cell-Free Silk Fibroin Biomaterial Strategy Promotes In Situ Cartilage Regeneration Via Programmed Releases of Bioactive Molecules. Adv. Healthc. Mater..

[37] Bossi A.M., Bucciarelli A., Maniglio D. (2021). Molecularly Imprinted Silk Fibroin Nanoparticles. ACS Appl. Mater. Interfaces.

[38] Kim S.H., Lee Y.J., Chao J.R., Kim D.Y., Sultan M.T., Lee H.J., Lee J.M., Lee J.S., Lee O.J., Hong H. (2020). Rapidly photocurable silk fibroin sealant for clinical applications. NPG Asia Mater..

[39] Zhou L., Wang Z., Chen D., Lin J., Li W., Guo S., Wu R., Zhao X., Lin T., Chen G. (2022). An injectable and photocurable methacrylate-silk fibroin hydrogel loaded with bFGF for spinal cord regeneration. Mater. Des..

[40] Pivar M., Gregor-Svetec D., Muck D. (2021). Effect of Printing Process Parameters on the Shape Transformation Capability of 3D Printed Structures. Polymers.

[41] Ai J.-R., Vogt B.D. (2022). Size and print path effects on mechanical properties of material extrusion 3D printed plastics. Prog. Addit. Manuf..

[42] Engler A.J., Sen S., Sweeney H.L., Discher D.E. (2006). Matrix Elasticity Directs Stem Cell Lineage Specification. Cell.

[43] Hengsberger S., Kulik A., Zysset P. (2002). Nanoindentation discriminates the elastic properties of individual human bone lamellae under dry and physiological conditions. Bone.

[44] Hong H., Lee O.J., Lee Y.J., Lee J.S., Ajiteru O., Lee H., Suh Y.J., Sultan M.T., Kim S.H., Park C.H. (2020). Cytocompatibility of Modified Silk Fibroin with Glycidyl Methacrylate for Tissue Engineering and Biomedical Applications. Biomolecules.

[45] Raggio R., Bonani W., Callone E., Dirè S., Gambari L., Grassi F., Motta A. (2018). Silk Fibroin Porous Scaffolds Loaded with a Slow-Releasing Hydrogen Sulfide Agent (GYY4137) for Applications of Tissue Engineering. ACS Biomater. Sci. Eng..

[46] Li S., Huang C., Liu H., Han X., Wang Z., Huang J., Yan Y., Wang Z. (2022). A Silk Fibroin Methacryloyl-Modified Hydrogel Promoting Cell Adhesion for Customized 3D Cell-Laden Structures. ACS Appl. Polym. Mater..

[47] Costa J.B., Silva-Correia J., Oliveira J.M., Reis R.L. (2017). Fast Setting Silk Fibroin Bioink for Bioprinting of Patient-Specific Memory-Shape Implants. Adv. Healthc. Mater..

[48] Wang J., Li X., Song Y., Su Q., Xiaohalati X., Yang W., Xu L., Cai B., Wang G., Wang Z. (2021). Injectable silk sericin scaffolds with programmable shape-memory property and neuro-differentiation-promoting activity for individualized brain repair of severe ischemic stroke. Bioact. Mater..

